# Functional Outcomes in Elderly Patients With Acute Coronary Syndrome: Conservative Versus Invasive Management

**DOI:** 10.7759/cureus.114902

**Published:** 2026-08-21

**Authors:** Jafin Basheer, Arun Thankappan, Ijas M Anakath, Gayathri Prathap, Gayathry Rajasekharan, Chinthu Sara Jacob, Rajasekharan Chandrasekharan

**Affiliations:** 1 Internal Medicine, Azeezia Institute of Medical Sciences and Research, Kollam, IND; 2 Internal Medicine, Sree Uthradom Thirunal Academy of Medical Sciences, Trivandrum, IND; 3 Pathology, Government Medical College, Thrissur, Thrissur, IND

**Keywords:** 7-item seattle angina questionnaire, acute coronary syndrome, cardiothoracic surgery, coronary artery bypass grafting, geriatrics and internal medicine, percutaneous coronary intervention, systemic hypertension

## Abstract

Introduction: In developing countries, cardiovascular disease is a major health burden, and there has been a rapid increase in the incidence of acute coronary syndrome (ACS) in the elderly population. Despite this, there are only limited studies or data available regarding the outcomes of ACS in elderly patients. Hence, this study focused on the management of ACS in the elderly treated conservatively and invasively.

Materials and methods: This longitudinal study was conducted at a tertiary care center in Kerala, India, involving 64 geriatric patients (aged 60 years and above) newly diagnosed with ACS. Subjects were recruited via convenience sampling and divided equally into invasive (percutaneous coronary intervention (PCI)/coronary artery bypass grafting (CABG); n = 32) and non-invasive/conservative treatment (n = 32) groups. Functional status was evaluated at initial admission, one month, and six months using the validated 7-item Seattle Angina Questionnaire (SAQ-7), which evaluates physical limitation, angina frequency, quality of life, and summary scores.

Results: At six months of follow-up, invasively treated patients had significantly better functional status in all domains compared to non-invasive patients (p = 0.001 for all domains). Systemic hypertension was present in 37 patients and was significantly associated with a poorer functional status summary score at six months (p = 0.001). No significant clear associations were noted for diabetes mellitus or dyslipidemia with a functional decline.

Conclusion: Geriatric patients presenting with ACS achieve substantially better functional capacity, reduced angina frequency, and superior health-related quality of life when managed with an invasive strategy compared to a conservative approach. Systemic hypertension acts as an independent risk factor for functional status decline.

## Introduction

Cardiovascular disease is a major public health burden worldwide and a leading cause of mortality, with the incidence of acute coronary syndrome (ACS) rising rapidly, particularly in developing countries [1,2]. Population aging has contributed to a shift from communicable diseases to non-communicable conditions, including atherothrombotic cardiovascular diseases [3]. Despite the growing number of elderly patients with ACS, evidence regarding their clinical outcomes and optimal management remains limited because older adults are often underrepresented in clinical trials [1].

This study aimed to compare the characteristics and functional status outcomes of elderly patients with ACS managed conservatively versus those treated with invasive strategies, including percutaneous coronary intervention (PCI) or coronary artery bypass grafting (CABG) [4]. In addition, the impact of major cardiovascular risk factors, including diabetes mellitus, hypertension, dyslipidemia, and adiposity, on functional recovery and long-term outcomes was evaluated [5-7]. Functional status was assessed using the validated 7-item Seattle Angina Questionnaire (SAQ-7), which evaluates physical limitation, angina frequency, quality of life, and overall health status [8]. In Kerala, one of the Indian states with the highest life expectancy and a rapidly aging population, the burden of ACS among older adults is increasing substantially. Although the Kerala ACS Registry has provided valuable information regarding the presentation, management patterns, and in-hospital outcomes of ACS patients, it primarily focused on mortality and major adverse cardiovascular events rather than patient-centered functional recovery following treatment [9]. Furthermore, data comparing functional outcomes between conservative and invasive management strategies specifically among elderly ACS patients in Kerala remains scarce. Given the unique demographic transition, high prevalence of cardiovascular risk factors, and differences in healthcare access within the state, there is a need for region-specific evidence evaluating how management strategies influence post-ACS functional status and quality of life in older adults. This study seeks to address this important knowledge gap by assessing functional outcomes using the SAQ-7 among elderly patients undergoing conservative versus invasive treatment approaches.

Baseline frailty, multimorbidity, and functional limitations may influence both treatment selection and subsequent recovery, potentially contributing to differences in outcomes between conservative and invasive strategies. Therefore, treatment-selection bias and residual confounding are important considerations when interpreting functional outcomes in observational studies of this population.

## Materials and methods

This longitudinal observational non-randomized study was conducted at Azeezia Institute of Medical Sciences and Research, in Kollam, Kerala, India, over an 18-month period starting in December 2019. Ethical approval was obtained from the Azeezia Ethics Committee (approval number: AEC/REV/2019/55), and written informed consent was obtained from all participants before enrolment.

A total of 64 patients aged ≥60 years with newly diagnosed ACS were recruited consecutively from inpatient and outpatient cardiology services using convenience sampling. Patients with a prior history of PCI or CABG, those unwilling to provide consent, and those unable to complete the study questionnaire were excluded.

Baseline data were collected using a structured proforma and included demographic details, clinical presentation, medical history, lifestyle habits, and cardiovascular risk factors. Clinical assessment included measurement of blood pressure, height, weight, body mass index (BMI), and waist-to-hip ratio.

Laboratory investigations included fasting blood glucose and lipid profile parameters.

ACS was diagnosed and classified as ST-segment elevation myocardial infarction (STEMI) or non-ST-segment elevation myocardial infarction (NSTEMI) based on electrocardiographic findings and cardiac biomarkers (troponin I), according to the American College of Cardiology/American Heart Association (ACC/AHA) guidelines [10]. Patients were managed either conservatively or with invasive revascularization (PCI/CABG) based on guideline recommendations and clinical judgment. Patients were managed according to the treating cardiologist's clinical assessment and the standard management strategy during the study period, and the non-invasive group comprised patients who did not undergo PCI or CABG.

Functional status was assessed at baseline, one month, and six months using the validated SAQ-7, which evaluates physical limitation, angina frequency, and quality of life. Permission to use the SAQ-7 was obtained from the copyright holder prior to study commencement, and the original source publication has been appropriately cited. The primary outcome was the change in functional status over time between the conservative and invasive treatment groups, while secondary outcomes included the associations of diabetes mellitus, systemic hypertension, dyslipidemia, and adiposity with functional recovery. Statistical analysis was performed using SPSS Statistics for Windows, V. 17.0 (SPSS Inc., Chicago, IL, USA).

## Results

Functional status outcomes at six months by treatment modalities

A total of 64 elderly patients with ACS were evaluated to compare six-month functional status outcomes between those managed conservatively (n = 32) and those who underwent invasive management (n = 32). Functional outcomes were measured using the self-administered SAQ-7.

Patients in the invasive management cohort demonstrated significantly higher scores across all evaluated SAQ domains compared to the conservative management cohort. The invasive group had higher scores for physical limitation (62.33 ± 18.92 vs. 23.44 ± 9.75), angina frequency (61.29 ± 18.72 vs. 27.60 ± 13.95), quality of life (65.31 ± 16.06 vs. 27.81 ± 12.11), and overall functional status (62.98 ± 16.67 vs. 26.28 ± 11.49). The differences between the two treatment pathways reached strong statistical significance across every domain (p = 0.001), indicating superior functional recovery, reduced symptom burden, and enhanced health-related quality of life among elderly patients receiving invasive interventions. The comprehensive six-month functional status outcomes and domain-specific comparisons between the conservative and invasive treatment groups are summarized in Table 1.

**Table 1:** Comparison of SAQ-7 domain scores between the non-invasive and invasive management groups Data are presented as mean ± SD. Comparisons between the non-invasive and invasive groups were performed using the independent samples Student's t-test. Mean differences are presented with 95% CI. A p-value of <0.05 was considered statistically significant. SAQ-7: 7-item Seattle Angina Questionnaire; SD: standard deviation; CI: confidence interval

SAQ-7 domain	Non-invasive (n = 32), mean ± SD	Invasive (n = 32), mean ± SD	Mean difference (95% CI)	t-statistic	P-value
Physical limitation	23.44 ± 9.75	62.33 ± 18.92	38.89 (31.37 to 46.41)	10.34	<0.001
Angina frequency	27.60 ± 13.95	61.29 ± 18.72	33.69 (25.44 to 41.94)	8.16	<0.001
Quality of life	27.81 ± 12.11	65.31 ± 16.06	37.50 (30.39 to 44.61)	10.55	<0.001
Overall SAQ-7 score	26.28 ± 11.49	62.98 ± 16.67	36.69 (29.54 to 43.85)	10.25	<0.001

Functional status severity at six months according to systemic hypertension

A significant association was observed between systemic hypertension and functional status at the six-month follow-up (p = 0.001). Specifically, 70.3% (n = 19/27) of patients without hypertension achieved good or excellent functional outcomes, whereas only 21.6% (n = 8/37) of hypertensive patients reached the same level. Conversely, a poor-to-fair functional status was overwhelmingly concentrated among hypertensive individuals at 78.4% (n = 29/37), compared to just 29.6% (n = 8/27) in the non-hypertensive group. Ultimately, these findings indicate that systemic hypertension is strongly linked to poorer long-term functional recovery following ACS, as summarized in Table 2.

**Table 2:** Functional status severity at six months according to systemic hypertension Distribution of functional status severity at six months according to the presence or absence of systemic hypertension. Data are presented as frequency count and percentage, formatted as n (%). The association between systemic hypertension and functional status severity was evaluated using the Pearson chi-squared test (degrees of freedom (df) = 2). A p-value of <0.05 was considered statistically significant; p = 0.001 indicates a highly significant statistical association.

	Systemic hypertension absent (n = 27)	Systemic hypertension present (n = 37)	Chi-squared value	P-value
Poor to fair	8 (29.6%)	29 (78.4%)	17.71	0.001
Good	10 (37.0%)	7 (18.9%)		
Excellent	9 (33.3%)	1 (2.7%)		
Total	27 (100.0%)	37 (100.0%)		

Change in functional status summary score from baseline to one month and six months in the invasively treated group

**Figure 1:**
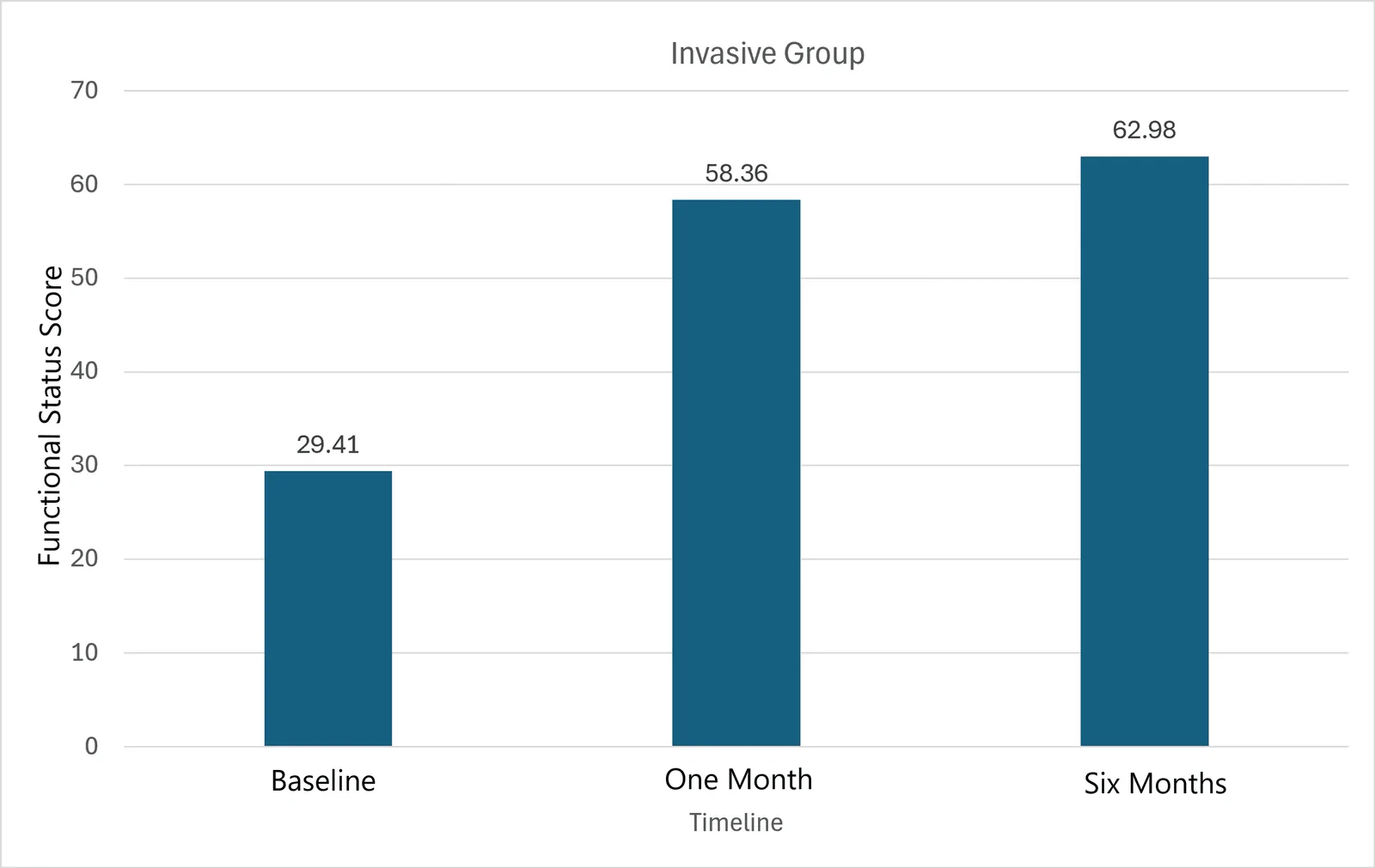
Change in functional status summary score from baseline to one month and six months in the invasively treated group Mean functional status scores in patients managed with the invasive strategy at baseline, one month, and six months. Functional status improved progressively from a mean score of 29.41 at baseline to 58.36 at one month and 62.98 at six months, demonstrating sustained functional recovery during follow-up.

A paired t-test was conducted to evaluate the changes in SAQ-7 scores over time. The analysis revealed a statistically significant improvement (p = 0.001) in mean SAQ-7 summary scores at both the one-month and six-month follow-ups compared to baseline among patients who underwent invasive treatment, as shown in Figure 1.

## Discussion

Our study was a longitudinal study for assessing the functional status outcomes in elderly ACS patients based on treatment modalities. Data were collected with the help of the validated SAQ-7 on initial admission pretreatment and at the one-month follow-up and the six-month follow-up during outpatient department visits.

As the elderly population grows in our country owing to our healthcare, the number of cases of ACS is also on the rise. Hence, the primary aim of our study was to identify the best treatment modality following which they could lead a basic, healthy, independent well-being as well as identify the risk factors which could hinder their functional status.

In the present study, the patient population comprised individuals aged 60 years and older, with a mean age of 69 years. The predominant age demographic was the 61-70-year-age group, accounting for 67.2% of the cohort. These demographic findings align with those reported by Kore et al., who observed a slightly higher mean age of 71 years [1]. Out of the total sample size of 64 patients analyzed in this study, 34 were male (53.1%), and 30 were female (46.9%).

Because the primary objective of this study was to assess and monitor functional status, the physical limitation domain was analyzed first. Patients managed with an invasive strategy (n = 32) demonstrated a mean physical limitation score of 56.34 at the one-month follow-up and 62.33 at the six-month follow-up. These values represented progressive mean increases of 28.37 and 34.36 from baseline, respectively, indicating favorable physical capacity and progressive improvement in performing daily activities.

Conversely, patients in the non-invasive (conservative) management cohort exhibited a mean score of 26.69 at one month and 23.44 at six months, reflecting a persistently poor-to-fair functional outcome. This difference between the two cohorts was statistically significant (p = 0.001), demonstrating substantially lower physical limitation among invasively treated patients. These findings closely align with those reported by Spertus et al., who similarly observed favorable outcome scores and a progressive mitigation of physical limitations following invasive cardiac procedures [11].

The second domain evaluated within the functional status assessment was angina frequency. In the invasive management cohort (n = 32), the mean angina frequency scores were 59.69 at the one-month follow-up and 61.29 at the six-month follow-up, representing substantial baseline increases of 29.88 and 31.48, respectively. These scores indicate a clinically meaningful reduction in anginal symptoms, with the majority of invasively treated patients experiencing symptoms less than weekly but at least once during the preceding month. Conversely, patients managed conservatively exhibited mean scores of 30.94 at one month and 27.60 at six months, a trajectory indicative of highly frequent symptoms occurring on a near-daily basis. This disparity between the treatment pathways was statistically significant (p = 0.001), demonstrating that invasive interventions effectively mitigate anginal symptom frequency over time.

These observations are consistent with a study by Zhang et al., which demonstrated substantial improvements in overall health status and a reduced angina burden predominantly among patients undergoing PCI and CABG [12]. In their cohort, the mean baseline angina frequency scores of 45.0 for PCI patients and 47.0 for CABG patients improved significantly, with mean increases ranging from 13.4 to 34.7 at six months and from 14.3 to 38.0 at 12 months.

Similar observations were reported by Cohen et al., who noted a significant reduction in angina burden at one, six, and 12 months of follow-up among invasively treated patients [13]. Specifically, PCI patients experienced an increase in angina frequency scores to 90.2, 91.1, and 92.7 from a baseline of 69.6. Similarly, patients undergoing CABG demonstrated improvements to 88.7, 92.7, and 93.8 from a baseline score of 69.5. These longitudinal improvements were found to be highly statistically significant (p < 0.05).

The third domain evaluated within the functional status assessment was health-related quality of life. Within the invasive management cohort (n = 32), the mean quality of life scores were 59.06 at the one-month follow-up and 65.31 at the six-month follow-up. In contrast, patients in the conservative management cohort experienced a downward trajectory, with mean quality of life scores of 32.81 at one month and 27.81 at six months. These lower values reflect a persistently poor-to-fair quality of life outcome. The markedly higher scores observed in the invasive treatment cohort reached statistical significance (p = 0.001), confirming a superior quality of life trajectory compared to conservative strategies.

These results align closely with previous literature. Cohen et al. [13] and Weintraub et al. [14] reported comparable findings, noting significant improvements in long-term health-related quality of life among ACS patients managed with early invasive strategies. Similarly, a study by Yang et al. evaluating quality of life outcomes in ACS patients treated via PCI or CABG demonstrated that revascularization conferred substantial, sustained benefits (p < 0.001) [15]. Over a six-month follow-up period, Yang et al. observed that quality of life scores increased from 40.0 ± 23.0 to 43.0 ± 17.0 in the PCI group, while scores in the CABG group improved from 48.0 ± 14.0 to 56.0 ± 11.0 [15].

Finally, the overall functional status score, reflecting a composite evaluation of the patient's well-being, was higher among elderly patients who underwent invasive management. In this cohort (n = 32), the mean overall functional status score was 58.36 at the end of the first month and increased to 62.98 by the sixth month of follow-up, representing a favorable functional status. Conversely, the conservative management cohort experienced a stagnant trajectory, with scores of 30.15 at one month and 26.28 at six months, indicating a persistently poor-to-fair functional capacity with no meaningful recovery from baseline. This distinct advantage favoring the invasive strategy reached high statistical significance (p = 0.001), confirming that invasive intervention translates into superior comprehensive functional recovery over time.

These statistical parameters align with contemporary literature evaluating elderly ACS cohorts. For instance, Kore et al. studied 233 elderly ACS patients and noted that the subsets undergoing PCI (n = 77) and CABG (n = 8) experienced superior outcomes compared to those allocated to medical management alone [1]. Similarly, Kunniardy et al. reviewed data from 1,052 ACS patients, where 9.4% of the cohort underwent an invasive procedure [16]. Their multivariate analysis identified an invasive strategy as the strongest independent predictor of both long-term survival and preserved functional status (p < 0.001). Furthermore, the After Eighty randomized controlled trial evaluated an elderly ACS cohort, demonstrating that an invasive management strategy significantly optimized clinical outcomes compared to conservative management. Over the follow-up period, patients assigned to the invasive group experienced markedly lower rates of repeat hospitalizations and adverse ischemic events than those managed non-invasively [17]. The primary comparison in this study was between conservative and invasive management strategies with respect to functional status outcomes at one and six months. Longitudinal changes from baseline were additionally assessed within the invasive management group to evaluate the trajectory of functional recovery over time.

Concurrently, a history of systemic hypertension was identified as a significant risk factor for adverse functional outcomes. Within the study cohort, 57.8% (n = 37/64) of patients had a documented history of systemic hypertension. Among these hypertensive individuals, 83.8% (n = 31/37) exhibited a poor-to-fair overall functional status score at the one-month follow-up, and 78.4% (n = 29/37) maintained this lower functional trajectory at the six-month follow-up. This correlation between pre-existing hypertension and diminished functional recovery reached high statistical significance (p = 0.001), establishing systemic hypertension as a strong predictor of poor functional status outcomes in elderly ACS patients. These findings closely parallel observations documented in the literature, including reports by Dumaine et al. [18] and Fresco et al. [19], which similarly highlighted the negative impact of hypertensive cardiovascular disease on long-term functional recovery post-ACS.

Regarding metabolic and lipid profiles, the study cohort included 54.7% (n = 35/64) of patients with a documented medical history of diabetes mellitus. Among these diabetic individuals, 68.6% (n = 24/35) exhibited a poor-to-fair overall functional status score at the one-month follow-up, and 60.0% (n = 21/35) maintained this lower functional trajectory at the six-month follow-up. Despite a notable proportion of diabetic patients experiencing diminished functional status, statistical analysis revealed no definitive, statistically significant association establishing diabetes mellitus as an independent risk factor for poor long-term functional recovery in this elderly ACS cohort [20].

A similar pattern was observed among patients with a history of dyslipidemia, who comprised 51.6% (n = 33/64) of the sample. In this subgroup, 72.7% (n = 24/33) demonstrated a poor-to-fair functional status score at one month, which decreased slightly to 63.6% (n = 21/33) at the six-month follow-up evaluation. Consistent with the diabetes findings, the statistical parameters analyzed in this study did not support a strong or statistically significant association between pre-existing dyslipidemia and a heightened risk of poor functional status outcomes post-ACS. These variations highlight the ongoing need to map distinct cardiovascular risk profiles across heterogeneous, vulnerable populations worldwide [21].

Overall, invasive management was associated with better SAQ-7-based functional status, angina control, and quality of life in elderly ACS patients over six months. However, given the observational, non-randomized design, these findings indicate association rather than causation and support an individualized, patient-centered approach to treatment.

This study has several limitations. It was a single-center, hospital-based study with a relatively small sample size (n = 64), which may limit the generalizability of the findings. Consecutive convenience sampling from a tertiary care center in Kerala may have introduced selection bias related to regional healthcare access. In addition, functional status was assessed using the self-administered SAQ-7 over a six-month follow-up period, making the findings susceptible to response bias and limiting the evaluation of long-term outcomes.

## Conclusions

This longitudinal study of 64 elderly ACS patients evaluated functional outcomes based on treatment modalities and identified risk factors for functional decline. Patients who underwent invasive management (PCI/CABG) demonstrated significantly better overall functional status, physical activity, angina control, and quality of life at both one- and six-month follow-ups compared to those managed conservatively. Consequently, invasive treatment in this population may reduce rehospitalizations, major adverse cardiac events, morbidity, and mortality. However, given the observational, non-randomized design, these findings indicate an association rather than a causal effect, and selection bias and residual confounding cannot be excluded.

Regarding risk factors, systemic hypertension was significantly associated with functional decline, emphasizing the need for strict blood pressure management through drug compliance and lifestyle modifications. While no significant associations were found for diabetes mellitus, dyslipidemia, or adiposity, larger studies are required to confirm these findings.
